# Assessment of Knowledge, Attitudes, and Practices Among Parents Regarding Antibiotic Use for Acute Respiratory Infections in Children: A Cross-Sectional Study

**DOI:** 10.7759/cureus.89849

**Published:** 2025-08-11

**Authors:** Mridula Dorairaj, Ramya R, Sundari S

**Affiliations:** 1 Department of Pediatrics, Sree Balaji Medical College and Hospital, Chennai, IND

**Keywords:** antibiotic use, knowledge-attitude-practice, pediatric health, public health education, acute respiratory infection

## Abstract

Background: Antibiotic misuse for childhood acute respiratory infections (ARIs) remains a major contributor to antimicrobial resistance, particularly in low- and middle-income countries. This study aimed to assess the knowledge, attitudes, and practices (KAP) of parents regarding antibiotic use for children with ARIs and to explore the influence of sociodemographic factors on KAP outcomes.

Methods: A cross-sectional study was conducted among 300 parents attending the Pediatrics Outpatient Department at Sree Balaji Medical College and Hospital, Chennai. Data were collected using a pre-validated structured questionnaire comprising items across knowledge, attitude, and practice domains. Descriptive statistics summarized participant characteristics and KAP scores. Inferential analyses, including chi-square tests, ANOVA, and bivariate regression, were used to assess associations between KAP scores and sociodemographic variables. Reliability and factor analyses validated the internal consistency and construct validity of the questionnaire.

Results: Mean scores indicated moderate levels of parental awareness and behavior regarding antibiotic use (Knowledge: 3.67; Attitude: 3.48; Practice: 3.84 on a five-point scale). Parental education, income, place of residence, and number of children were significantly associated with KAP scores (p < 0.05). Parents with fewer children, higher education levels, lower income, and urban residence demonstrated significantly better knowledge and practices. A strong positive correlation was observed between knowledge and practice domains (R = 0.511, p < 0.001), indicating that informed parents were more likely to adopt appropriate antibiotic use behaviors. ANOVA revealed significant differences in KAP scores across different demographic subgroups.

Conclusion: The findings highlight the urgent need for targeted health education programs to improve antibiotic-related knowledge and practices, particularly in rural, less-educated, and higher-income populations. Promoting rational antibiotic use among parents may help to curb the growing threat of antimicrobial resistance. Future research should evaluate the impact of educational interventions across diverse communities.

## Introduction

Among the most significant medical developments of the 20th century was the creation of antibiotics, which are the most prevalent category of medicines. Millions of lives have been saved, and human civilization has profited from the discovery of antibiotics in the fight against microorganisms [1]. Antibiotic resistance, however, has become a significant global public health concern in recent years [2]. The treatment of common illnesses is hampered by this increasing resistance, which raises death rates, prolongs sickness, and raises healthcare expenses [3]. Antibiotic-resistant diseases are thought to be the cause of over 700,000 fatalities every year [4].

Antibiotic resistance is particularly concerning in the context of pediatric illnesses, where certain infections often prompt the overuse of antibiotics. Acute respiratory infections (ARIs) are among the most prevalent of these conditions and are a major contributor to pediatric morbidity and death worldwide. In low-income nations, ARIs are responsible for over one-third of mortality among children under five [5]. Both upper and lower respiratory tract infections are considered ARIs; the most prevalent ARIs are influenza and the common cold [6]. Short, fast breathing or chest-related breathing difficulties are signs of ARI. About 15% of all pediatric fatalities worldwide are caused by pneumonia, a manifestation of ARI [7]. While the majority of these ARIs are caused by viral pathogens, antibiotics are frequently demanded by caregivers. Even though it is known that antibiotics are useless against viral infections, this tendency still exists, resulting in needless treatments and possible health risks for the child. This excessive demand is exacerbated by misplaced parental expectations and the accessibility of over-the-counter antibiotics. Antibiotics are often purchased from pharmacies or unofficial sellers in India without a prescription, which leads to a high rate of self-medication [8]. According to reports, more than half of antibiotics are obtained without a prescription, highlighting a serious regulatory control vacuum [9].

The general populace in India still lacks enough knowledge and comprehension of how to take antibiotics appropriately [10]. Perceptions and behaviors around the use of antibiotics are significantly influenced by socioeconomic variables [8]. Antibiotic abuse, such as using them without a doctor's prescription and not finishing the whole course of treatment, has increased significantly. These behaviors demonstrate the critical need for public health initiatives that encourage prudent antibiotic use, especially among parents of small children who often use these drugs.

Research shows that parents' knowledge, attitudes, and practices (KAP) about antibiotics are often deficient and heavily impacted by factors such as family poverty and educational background [11,12]. Due to their increased vulnerability to infectious infections, children, particularly those under five, are prescribed a significant amount of antibiotics. On the other hand, these drugs are not usually taken as prescribed [13].

Among Indian parents, a number of myths are common. Many people think that antibiotics should be taken anytime a kid develops a fever or that they are beneficial against viral illnesses. Some people believe that shorter antibiotic courses are better for their child's health [11]. These misconceptions lead to the overuse and abuse of antibiotics, especially when treating ARIs, which are among the most prevalent illnesses affecting children in India.

Many factors contribute to the improper use of antibiotics. Due to a lack of diagnostic resources, worries about patient follow-up, or pressure from caregivers, medical professionals may prescribe antibiotics in advance in many impoverished or rural locations [10]. Doctors may give antibiotics even when they are not medically essential due to parental pressure and expectations [14]. This reflects a broader concern regarding the KAP among parents, where limited understanding, inappropriate beliefs, and poor decision-making behaviors combine to perpetuate antibiotic misuse. This KAP gap emphasizes the need for focused initiatives to educate caregivers on the proper use of antibiotics, especially when it comes to viral infections.

Research particularly evaluating parental KAP addressing the use of antibiotics for pediatric illnesses is lacking in India, despite the importance of this topic. Understanding parental behavior and expertise in this area is essential, given the growth of the population and the high prevalence of infectious illnesses. The purpose of this study is to evaluate Indian parents of children under five years old's knowledge, attitudes, and behaviors about the use of antibiotics in the treatment of ARIs.

## Materials and methods

Study design and setting

A cross-sectional study design was chosen for its effectiveness in capturing KAP data at a single point in time across a defined population. The setting for the study was the Pediatric OPD of a tertiary care hospital, Sree Balaji Medical College and Hospital, in Chennai. This location was selected due to its high daily patient turnover and its accessibility to families from various socioeconomic and educational backgrounds. The study was conducted over a period of three months (January to March 2025), during which data were collected both in person and online to maximize participation and reach.

Study population 

The study targeted parents of children aged zero to 14 years who were seeking treatment for ARIs. This age range was selected to include parents across various stages of childhood dependency, ensuring the relevance of parental decision-making in antibiotic use. Parents who met the inclusion criteria were approached and invited to participate. The inclusion criteria included being the parent or guardian of a child aged zero to 14 years, visiting the Pediatric OPD for ARI-related symptoms, and providing informed consent. Parents with medical or paramedical training, those who were unable to understand what antibiotics are, those not willing to participate, or parents of children with chronic illnesses or undergoing long-term steroid therapy were excluded from the study.

Sample size

The sample size was determined based on a prior study by Hassan BAR et al. [15], which found that 56.14% of parents had adequate knowledge of antibiotic use for upper respiratory infections. Using a 95% confidence interval and 10% relative precision, the sample size was calculated, and a total of 300 parents were enrolled. A purposive sampling technique was used to select participants, as the study required input from a specific population, parents of children with ARIs.

Data collection

Primary data were collected through a structured, pretested questionnaire that was administered in both English and Tamil to ensure comprehension across educational levels (Appendix 1). The questionnaire was divided into three major sections corresponding to the study variables: knowledge, attitude, and practice. The knowledge section included multiple-choice questions that assessed awareness of the purpose, effects, and appropriate use of antibiotics. The attitude section used Likert-scale statements to gauge respondents' beliefs and confidence in physician guidance, trust in antibiotics, and perceptions about their necessity for viral infections. The practice section documented actual behaviors, such as adherence to prescriptions, self-medication tendencies, and storage and reuse of leftover antibiotics.

To improve reach, especially among urban and semi-urban populations, data collection was carried out both in person and via digital platforms such as Google Forms (Google LLC, Mountain View, California, United States) and email. Participants were also encouraged to reflect on their most recent experiences with antibiotic use for their children. In addition to primary data, secondary sources like hospital pediatric health records and community-level public health guidelines were reviewed to provide context and validate self-reported behaviors.

The research tool was a structured questionnaire that underwent both face and content validation before full deployment. The instrument was divided into sections on knowledge, attitude, and practice. Each section generated a composite score to quantify the respondent’s position in each domain. Knowledge was operationally defined as adequate if the participant answered at least 70% of knowledge-related items correctly. A favorable attitude was defined as a mean score greater than 3.5 on a 5-point Likert scale. Appropriate practice was defined by behavior such as never self-initiating antibiotics, always completing the prescribed course, and avoiding reuse of previously prescribed antibiotics. 

This study employed exploratory factor analysis (EFA) to validate the 15-item tool assessing knowledge, attitude, and practice. Factors with eigenvalues > 1 were extracted using the Kaiser criterion [16]. Varimax rotation with Kaiser normalization was applied, and items with loadings ≥ 0.40 were retained. As the tool was newly developed, EFA was appropriate to explore latent constructs. Demographic variables, including age, sex, education, monthly income, number of children, and place of residence, were recorded as explanatory variables to explore associations with KAP levels. These were included in the final analysis to identify predictive trends and areas for targeted intervention.

Ethical considerations

Prior to data collection, ethical clearance was obtained from the Institutional Ethics Committee of Sree Balaji Medical College and Hospital (002/SBMCH/IHEC/2025/2346). Participation was entirely voluntary, and informed consent was obtained from all respondents. Participants were assured of the anonymity and confidentiality of their responses. The study posed minimal risk, involving non-invasive data collection and adhering to the ethical guidelines provided by the Council for International Organizations of Medical Sciences.

Statistical analysis

Data were entered into Microsoft Excel (Microsoft Corporation, Redmond, Washington, United States) and analyzed using IBM SPSS Statistics for Windows, Version 26 (Released 2018; IBM Corp., Armonk, New York, United States). Descriptive statistics, including means, frequencies, and percentages, were calculated for demographic characteristics and KAP scores. Chi-square tests were used to examine associations between categorical variables and KAP outcomes. ANOVA was applied to detect significant differences in mean KAP scores across demographic groups. The Pearson correlation coefficient was used to look into the correlation between knowledge and practice. Factor analysis and Cronbach’s alpha were used to assess construct validity and internal consistency, respectively, of the KAP questionnaire. A p-value < 0.05 was considered statistically significant.

## Results

In this study involving 300 parents, the majority (124; 41.3%) were aged > 18-25 years, followed by 76 (25.3%) in the 26-35 years range. There were 166 males (55.3%) and 134 females (44.7%). Regarding educational background, 80 (26.7%) had no formal education, 65 (21.7%) had primary education, and 75 (25%) had postgraduate qualifications. Among occupations, 108 (36%) were homemakers, while 80 (26.7%) were employed in government jobs. Most participants (148; 49.3%) had two children. The age of children was fairly distributed, with 93 (31%) being below one year and 73 (24.3%) aged seven years and above. Family structure was equally divided, with 120 (40%) each living in nuclear and joint families and 60 (20%) in extended families. In terms of place of residence, 144 (48%) were from semi-urban areas, 84 (28%) from urban, and 72 (24%) from rural settings. Monthly income distribution showed 80 (26.7%) earning below ₹10,000 and 75 (25%) earning above ₹30,000. Notably, 210 (70%) reported having received some form of health education, indicating a relatively informed sample (Table 1).

**Table 1:** Sociodemographic characteristics of study participants (n = 300) * Prior health education about antibiotics

Variables	Groups	Frequency (n)	Percent (%)
Parent Age	>18–25 years	124	41.3
	26–35 years	76	25.3
	36–45 years	52	17.3
	46 years and above	48	16.0
Gender	Male	166	55.3
	Female	134	44.7
Parent Education Level	No formal education	80	26.7
	Primary	65	21.7
	Secondary	50	16.7
	Graduate	30	10.0
	Postgraduate	75	25.0
Occupation	Homemaker	108	36.0
	Self-employed	21	7.0
	Private job	25	8.3
	Government job	80	26.7
	Others	66	22.0
Number of Children	One child	72	24.0
	Two children	148	49.3
	Three or more children	80	26.7
Age of Child	Below 1 year	93	31.0
	1–3 years	66	22.0
	4–6 years	68	22.7
	7 years and above	73	24.3
Type of Family	Nuclear	120	40.0
	Joint	120	40.0
	Extended	60	20.0
Place of Residence	Urban	84	28.0
	Semi-urban	144	48.0
	Rural	72	24.0
Monthly Income	Below ₹10,000	80	26.7
	₹10,001–20,000	76	25.3
	₹20,001–30,000	69	23.0
	Above ₹30,000	75	25.0
Health Education*	Yes	210	70.0
	No	90	30.0

Among the 300 respondents, the mean scores for knowledge, attitude, and practice regarding antibiotic use for childhood ARIs were 3.67 ± 0.75, 3.48 ± 0.69, and 3.84 ± 0.77, respectively. The minimum and maximum scores ranged from 1.00 and 5.00.

To evaluate the suitability of data for factor analysis, the Kaiser-Meyer-Olkin (KMO) measure and Bartlett’s test of sphericity were conducted. The KMO value was 0.890, indicating excellent sampling adequacy for factor analysis. Bartlett’s test yielded a chi-square of 2737.226 (df = 105, p < 0.001), confirming that the correlation matrix is significantly different from an identity matrix. These results together affirm that the dataset was appropriate for further factor extraction. Principal component analysis with varimax rotation was employed to extract the underlying dimensions of the questionnaire. Communality values for all 15 variables exceeded 0.5, ranging from 0.530 (Attitude Statement 3, Appendix 1) to 0.814 (Practice Statement 5, Appendix 1), demonstrating that each item shared a substantial amount of common variance with other items in the dataset and hence was retained for further analysis (Table 2). Reliability testing revealed excellent internal consistency for all constructs. Cronbach’s alpha ranged from 0.851 (attitude) to 0.926 (practice), and average variance extracted (AVE) and composite reliability (CR) values exceeded recommended thresholds, confirming the scales’ validity (Table 3).

**Table 2:** Validity of the questionnaire used by factor analysis

Components	Initial Eigenvalues	% of Variance	Cumulative %
Knowledge	6.361	42.409	42.409
Attitude	2.413	16.087	58.496
Practice	1.687	11.249	69.745

**Table 3:** Reliability of the questionnaire AVE: average variance extracted; CR: composite reliability

Components	Cronbach Alpha Value	AVE	CR
Knowledge	0.872	0.682	0.829
Attitude	0.851	0.634	0.808
Practice	0.926	0.775	0.862

Table 4 summarizes the mean knowledge scores across sociodemographic groups. Higher knowledge was observed among younger parents (18-25 years: M = 3.85 ± 0.62), females (M = 3.70 ± 0.76), and those with no formal education (M = 3.97 ± 0.60) or postgraduate degrees (M = 3.83 ± 0.59). Government employees (M = 3.80 ± 0.71) and homemakers (M = 3.70 ± 0.72) also showed better knowledge. Parents with one child (M = 4.01 ± 0.59), urban residents (M = 3.94 ± 0.62), and those with income below ₹10,000 (M = 3.98 ± 0.59) had higher scores. Additionally, those who received health education reported better knowledge (M = 3.80 ± 0.66).

**Table 4:** Association of mean knowledge scores across sociodemographic variables (n = 300) * p-value < 0.05 is significant

Sociodemographic Variable	Category	Mean ± SD	F-value	p-value
Age of Parent	18–25 years	3.85 ± 0.62	6.21	<0.001*
	26–35 years	3.70 ± 0.65		
	36–45 years	3.60 ± 0.70		
	46 years and above	3.40 ± 0.82		
Gender	Male	3.65 ± 0.75	1.02	0.314
	Female	3.70 ± 0.76		
Education Level	No formal education	3.97 ± 0.60	15.83	<0.001*
	Primary	3.55 ± 0.66		
	Secondary	3.60 ± 0.73		
	Graduate	2.86 ± 1.04		
	Postgraduate	3.83 ± 0.59		
Occupation	Homemaker	3.70 ± 0.72	4.56	<0.001*
	Self-employed	3.60 ± 0.65		
	Private job	3.50 ± 0.64		
	Government job	3.80 ± 0.71		
	Others	3.65 ± 0.68		
Number of Children	One child	4.01 ± 0.59	24.62	<0.001*
	Two children	3.74 ± 0.62		
	≥ Three children	3.24 ± 0.90		
Child's Age	Below 1 year	3.80 ± 0.63	5.87	<0.001*
	1–3 years	3.75 ± 0.65		
	4–6 years	3.60 ± 0.68		
	≥ 7 years	3.40 ± 0.88		
Type of Family	Nuclear	3.72 ± 0.70	2.11	0.122
	Joint	3.68 ± 0.74		
	Extended	3.60 ± 0.68		
Place of Residence	Urban	3.94 ± 0.62	20.92	<0.001*
	Semi-urban	3.74 ± 0.62		
	Rural	3.23 ± 0.93		
Monthly Income	< ₹10,000	3.98 ± 0.59	14.92	<0.001*
	₹10,001–20,000	3.68 ± 0.62		
	₹20,001–30,000	3.78 ± 0.62		
	> ₹30,000	3.24 ± 0.93		
Health Education	Yes	3.80 ± 0.66	11.45	<0.001*
	No	3.45 ± 0.84		

The attitude scores varied across different sociodemographic groups. Parents aged 18-25 years had the highest mean attitude score (3.60 ±​​​​​​ 0.68), while those aged 46 and above had the lowest (3.30 ± 0.74). Female participants scored slightly higher (3.52 ± 0.68) than males (3.45 ± 0.71). Among education levels, both those with no formal education and postgraduates scored highest (3.60 ± 0.71), whereas graduates scored lowest (3.13 ± 0.86). Government employees and homemakers had relatively high attitude scores, while private job holders had lower scores (3.35 ± 0.74). Parents with only one child showed the most favourable attitudes (3.60 ± 0.72), and those with three or more children the least (3.21 ± 0.78). Urban and semi-urban residents reported higher attitude scores compared to rural residents. Attitude scores were also higher among those with lower income and those who received health education (Table 5).

**Table 5:** Association of mean attitude scores across sociodemographic variables (n = 300) * p-value < 0.05 is significant

Sociodemographic Variable	Category	Mean ± SD	F-value	p-value
Age of Parent	18–25 years	3.60 ± 0.68	2.213	0.086
	26–35 years	3.50 ± 0.66		
	36–45 years	3.40 ± 0.71		
	46 years and above	3.30 ± 0.74		
Gender	Male	3.45 ± 0.71	1.245	0.265
	Female	3.52 ± 0.68		
Education Level	No formal education	3.60 ± 0.71	3.492	0.008
	Primary	3.43 ± 0.53		
	Secondary	3.39 ± 0.75		
	Graduate	3.13 ± 0.86		
	Postgraduate	3.60 ± 0.66		
Occupation	Homemaker	3.50 ± 0.69	2.014	0.092
	Self-employed	3.40 ± 0.67		
	Private job	3.35 ± 0.74		
	Government job	3.60 ± 0.65		
	Others	3.45 ± 0.72		
Number of Children	One child	3.60 ± 0.72	9.088	<0.001*
	Two children	3.57 ± 0.59		
	≥ Three children	3.21 ± 0.78		
Child's Age	Below 1 year	3.65 ± 0.61	2.342	0.073
	1–3 years	3.50 ± 0.68		
	4–6 years	3.45 ± 0.74		
	≥ 7 years	3.30 ± 0.77		
Type of Family	Nuclear	3.55 ± 0.69	1.673	0.189
	Joint	3.48 ± 0.70		
	Extended	3.40 ± 0.73		
Place of Residence	Urban	3.58 ± 0.70	8.279	<0.001*
	Semi-urban	3.57 ± 0.60		
	Rural	3.20 ± 0.81		
Monthly Income	< ₹10,000	3.61 ± 0.70	4.814	0.003*
	₹10,001–20,000	3.52 ± 0.54		
	₹20,001–30,000	3.57 ± 0.66		
	> ₹30,000	3.23 ± 0.82		
Health Education	Yes	3.60 ± 0.64	14.312	<0.001*
	No	3.25 ± 0.72		

Practice scores varied notably across several sociodemographic groups. Higher mean scores were observed among parents aged 18-25 years (4.00 ± 0.61), females (3.90 ± 0.69), those with no formal education (4.16 ± 0.61), government employees (3.95 ± 0.65), and those with one child (4.13 ± 0.61). Urban residents (4.07 ± 0.62), lower-income groups (4.09 ± 0.64), and those who received health education (3.95 ± 0.68) also reported significantly better practices. These differences were statistically significant across age, education, occupation, number of children, child’s age, place of residence, income, and health education status (Table 6).

**Table 6:** Association of mean practice scores across sociodemographic variables (n = 300) * p-value < 0.05 is significant

Sociodemographic Variable	Category	Mean ± SD	F-value	p-value
Age of Parent	18–25 years	4.00 ± 0.61	2.723	0.045
	26–35 years	3.85 ± 0.65		
	36–45 years	3.70 ± 0.73		
	46 years and above	3.50 ± 0.78		
Gender	Male	3.78 ± 0.73	1.492	0.223
	Female	3.90 ± 0.69		
Education Level	No formal education	4.16 ± 0.61	8.882	<0.001*
	Primary	3.67 ± 0.68		
	Secondary	3.79 ± 0.86		
	Graduate	3.29 ± 0.98		
	Postgraduate	3.89 ± 0.71		
Occupation	Homemaker	3.90 ± 0.72	2.539	0.041*
	Self-employed	3.70 ± 0.73		
	Private job	3.60 ± 0.76		
	Government job	3.95 ± 0.65		
	Others	3.75 ± 0.74		
Number of Children	One child	4.13 ± 0.61	13.265	<0.001*
	Two children	3.87 ± 0.73		
	≥ Three children	3.51 ± 0.87		
Child's Age	Below 1 year	4.05 ± 0.60	2.891	0.036*
	1–3 years	3.80 ± 0.71		
	4–6 years	3.75 ± 0.75		
	≥ 7 years	3.60 ± 0.79		
Type of Family	Nuclear	3.90 ± 0.69	1.827	0.163
	Joint	3.82 ± 0.71		
	Extended	3.70 ± 0.76		
Place of Residence	Urban	4.07 ± 0.62	11.751	<0.001*
	Semi-urban	3.87 ± 0.73		
	Rural	3.50 ± 0.90		
Monthly Income	< ₹10,000	4.09 ± 0.64	7.401	<0.001*
	₹10,001–20,000	3.78 ± 0.68		
	₹20,001–30,000	3.94 ± 0.75		
	> ₹30,000	3.54 ± 0.92		
Health Education	Yes	3.95 ± 0.68	10.986	<0.001*
	No	3.60 ± 0.75		

**Figure 1:**
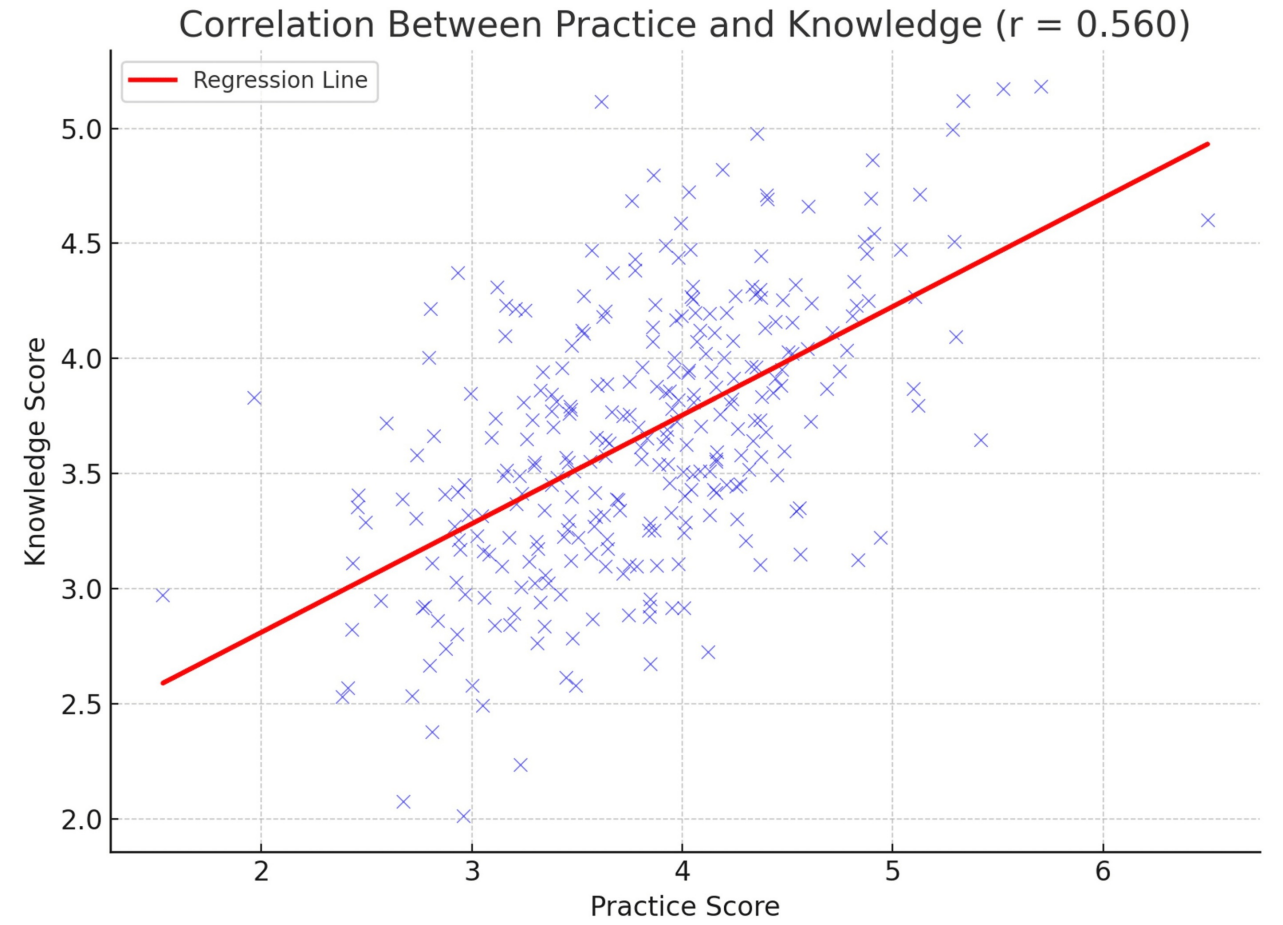
Correlation between practice and knowledge of the study participants towards antibiotic use in the pediatric population

The analysis revealed a moderate positive relationship between parental knowledge and practice concerning antibiotic use for childhood ARIs. The correlation coefficient (R) was found to be 0.560, indicating that as parents engage in more appropriate antibiotic practices, their knowledge levels tend to be higher. Furthermore, the R-square value of 0.261 suggests that approximately 26.1% of the variability in knowledge scores can be explained by their reported practice behaviors. This underscores the importance of practice-based learning and behavior reinforcement in improving health literacy among parents (Figure 1).

## Discussion

This study assessed the KAP among parents regarding antibiotic use for ARIs in children at a tertiary care hospital in Chennai, India. Our findings revealed that although many parents demonstrated a high self-reported rate of antibiotic use for ARI symptoms, their knowledge regarding appropriate indications was limited. A considerable proportion of parents believed that antibiotics could treat minor viral illnesses such as colds, flu, headaches, and body aches, reflecting a widespread misconception also noted in previous literature. The tendency to self-medicate with antibiotics for such viral conditions is a growing concern, which contributes significantly to AMR [17].

Inappropriate antibiotic use was largely driven by misinformation and easy access. A substantial number of parents reported using antibiotics without prescriptions, often obtained from leftover medications or purchased directly from pharmacies. This aligns with findings by Abood et al. (2018), who highlighted the impact of unregulated over-the-counter antibiotic sales in promoting improper medication practices [18]. Beyond licensed healthcare providers, the study identified non-professional sources such as the internet, media, and social networks as common channels through which parents obtained information about antibiotics and resistance. This raises concerns about the dissemination of inaccurate information, consistent with observations made by Schifano et al. (2021) and Marathe et al. (2020) [19,20].

Another key observation was the role of healthcare providers in guiding appropriate antibiotic use. Some participants reported receiving inadequate instruction from medical professionals regarding antibiotic usage, which led to misuse. This underscores the need for healthcare personnel to strengthen their patient education efforts, a recommendation echoed in studies by Hoppe et al. (2020) and Polasek et al. (2019) [21,22]. While patients must ultimately be responsible for adhering to treatment guidelines, clear and consistent communication from clinicians remains vital.

The study also revealed limited awareness among parents regarding AMR and its health consequences. Many participants appeared unaware that misuse of antibiotics could lead to resistance, which could, in turn, render treatment ineffective for more serious conditions, including cancer and post-surgical infections. Furthermore, some believed that AMR only affects individuals who take antibiotics frequently, a misconception debunked in studies by Thorpe et al. and Meerza et al. [23,24]. These perceptions reduce the urgency of adopting rational antibiotic practices.

A prevalent behavior identified in the study was the premature discontinuation of antibiotics once children began feeling better. This incomplete treatment approach was noted as a major contributor to AMR, aligning with global findings [25,26]. The sharing of leftover antibiotics with others further exacerbates the risk. The study also established a significant positive correlation between knowledge and practice, indicating that parents with higher knowledge scores were more likely to engage in appropriate antibiotic use. These findings align with earlier literature suggesting that educational interventions and improved access to accurate medical information are crucial in addressing misconceptions and misuse [27].

Limitations

This study was conducted in a single tertiary care hospital in Chennai, which may limit the generalizability of the findings to other populations. Data were self-reported, which may introduce recall and social desirability biases. Additionally, the study did not explore healthcare providers' perspectives, which could offer further insight into prescription practices and communication barriers. Future studies involving multi-center data and including perspectives from clinicians and pharmacists would enhance the understanding of antibiotic use behaviors among parents in India.

## Conclusions

This study highlights significant associations between parental sociodemographic factors and KAP regarding antibiotic use for childhood ARIs. Education level, number of children, income, and place of residence were all found to significantly influence KAP scores. Interestingly, lower-income and less formally educated parents often reported better practices, possibly reflecting greater reliance on public health messaging or more cautious behavior. Additionally, a moderate positive correlation was observed between knowledge and practice, indicating that improved awareness may lead to more appropriate antibiotic use. These findings underscore the need for targeted educational interventions, particularly in rural and higher-income urban settings, to ensure rational antibiotic use and help combat antimicrobial resistance.

## Appendices

Appendix 1 

**Table 7:** Knowledge, attitudes, and practices (KAP) questionnaire in assessing the antibiotic usage in children

Domain	Statements	Strongly Disagree	Disagree	Neutral	Agree	Strongly Agree
Knowledge	Antibiotics are effective only against bacterial infections.	☐	☐	☐	☐	☐
	Antibiotics are not effective for viral infections such as the common cold.	☐	☐	☐	☐	☐
	Overuse of antibiotics can lead to antibiotic resistance.	☐	☐	☐	☐	☐
	Correct dosage and duration of antibiotics are essential for effective treatment.	☐	☐	☐	☐	☐
	Viral and bacterial infections require different treatments.	☐	☐	☐	☐	☐
Attitude	Antibiotics should be given for every episode of fever.	☐	☐	☐	☐	☐
	Doctors’ decisions regarding antibiotic prescriptions are trustworthy.	☐	☐	☐	☐	☐
	Antibiotics should be used only when absolutely necessary.	☐	☐	☐	☐	☐
	Demanding antibiotics during minor illnesses is inappropriate.	☐	☐	☐	☐	☐
	Completing the full course of antibiotics is important even if symptoms improve.	☐	☐	☐	☐	☐
Practice	Doctor’s instructions are always followed when administering antibiotics.	☐	☐	☐	☐	☐
	Antibiotics are not used without a doctor’s prescription.	☐	☐	☐	☐	☐
	Leftover antibiotics are sometimes used for new illnesses.	☐	☐	☐	☐	☐
	Antibiotic treatment is stopped once the symptoms subside.	☐	☐	☐	☐	☐
	Antibiotics are kept at home for future use without medical advice.	☐	☐	☐	☐	☐
